# Metabolic impact of switching from EFV/TDF/FTC to two INSTI-Based regimens (B/F/TAF vs. DTG/3TC): a real-world study in virologically suppressed individuals

**DOI:** 10.3389/fphar.2026.1753242

**Published:** 2026-03-31

**Authors:** Er Li, Yi Wang, Dingyan Yan, Jinchuan Shi, Jianhua Yu, Xiaowei Hu, Wenhui Zhang

**Affiliations:** 1 Affiliated Hangzhou Xixi Hospital, Zhejiang Chinese Medical University, Hangzhou, China; 2 Xihu District Center for Disease Control and Prevention, Hangzhou, China

**Keywords:** antiretroviral therapy, HIV, metabolic safety, real-world evidence, regimen switch, weight gain

## Abstract

**Objective:**

To compare metabolic outcomes in virologically suppressed PLWH switching from EFV/TDF/3TC to either B/F/TAF or DTG/3TC.

**Methods:**

In this retrospective real-world study of 326 patients, weight, lipid (TG, TC), glucose (GLU), and uric acid (SUA) changes were assessed over 24 months. Generalized linear mixed models and logistic regression identified factors associated with metabolic trends and clinically significant weight gain (>5%).

**Results:**

The B/F/TAF and DTG/3TC groups showed no significant between-group differences in the longitudinal trends of TG, TC, GLU, or SUA, the incidence of metabolic abnormalities, or the risk of >5% weight gain (all *P* > 0.05). Within-group analyses indicated the B/F/TAF regimen increased TC (*P* < 0.001) and decreased GLU (*P* = 0.013), whereas DTG/3TC did not significantly alter TG, TC, or GLU levels. Both regimens increased SUA and body weight (B/F/TAF: +2.5 kg; DTG/3TC: +3.3 kg; both *P* < 0.001). Modifiable risk factors were associated with metabolic outcomes: smoking with increased TG and TC; hypertension and BMI≥25 kg/m^2^ with increased TC, GLU, and SUA; age≥50 years with increased GLU and SUA; and regular alcohol consumption with >5% weight gain (adjusted OR = 1.92, *P* = 0.008). These findings remained consistent in participants with hypertension.

**Conclusion:**

In virologically suppressed PLWH, switching to B/F/TAF or DTG/3TC provides comparable metabolic safety profiles. Clinical management should prioritize individualized regimen selection alongside proactive management of traditional metabolic risk factors.

## Introduction

Antiretroviral therapy (ART) is the cornerstone of HIV management, enabling long-term viral suppression and immune recovery (Patel et al., 2021; Lee et al., 2024). However, ART also entails cumulative and evolving metabolic, cardiovascular, and body composition effects that influence the long-term health of people living with HIV (PLWH). Weight gain is recognized as clinically significant and associated with adverse metabolic outcomes, including dyslipidemia, insulin resistance, and an increased risk of cardiovascular disease (CVD) which is the main cause of non-acquired immunodeficiency syndrome (AIDS)-related mortality (Kumar and Samaras, 2018; Ryan et al., 2025; Bijker et al., 2019; Bijker et al., 2017). Serum uric acid (SUA), blood glucose (GLU) and lipid levels serve as valuable biomarkers for detecting and preventing the early onset of CVD (Kimura et al., 2021; Li et al., 2023; Chiang et al., 2019). Hyperuricemia occurs earlier than hyperlipidemia and diabetes, suggesting that elevated SUA plays an upstream role in the occurrence and development of CVDs (Priscilla et al., 2021).

Hyperuricemia (HUA) and dyslipidemia are common in PLWH on maintenance therapy (Liu et al., 2021; Wenjing et al., 2025). An Italian study reported a 25.2% prevalence of HUA among PLWH following long-term antiretroviral therapy (ART) (Pirro et al., 2018). SUA levels are increased by dolutegravir-, stavudine-, didanosine-, or protease inhibitor-based regimens, reduced by tenofovir, and unaffected by abacavir (Hongo et al., 2021; Fernandes et al., 2023; Alter et al., 2006). A recent Chinese systematic review highlighted the uncertain impact of ART regimens on lipid profiles, as shown by LPV/r-based regimens having the highest prevalence of high TG (followed by INSTI- and EFV-based regimens), despite the lack of statistical significance in these differences. Furthermore, identified risk factors (e.g., high body mass index [BMI]) further complicate the relationship (Gan et al., 2023).

Currently, dolutegravir (DTG) and bictegravir (B) are the most commonly used integrase inhibitors for PLWH, with a higher barrier to resistance, better efficacy profiles, better safety profiles and fewer drug-drug interactions compared with other integrase inhibitors (Venter et al., 2019; Gandhi et al., 2023). Thus, DTG- or B-based regimens are recommended for the initial therapy and switch therapy in PLWH (Gan et al., 2023; Venter et al., 2019). In China, the EFV/tenofovir disoproxil fumarate (TDF)/lamivudine (3TC) with cumulative toxicity regimen was once a widely used first-line regimen within the national free antiretroviral treatment program (Wei et al., 2023). Consequently, there is a substantial population of virologically suppressed PLWH still maintained on this regimen. Switching optimized INSTI-based regimens (such as DTG/3TC or B/F/TAF) to EFV/TDF/3TC, because of pill burden or food restrictions, risk of drug interactions, toxicity, or negative effects on age-related comorbidities, is becoming increasingly important. Despite B/F/TAF have been associated with greater weight gain compared to DTG/3TC, no fully trials have compared them over the long term (Ryan et al., 2025).

This study aimed to retrospectively evaluate the safety and associated factors of switching regimens to DTG/3TC versus B/F/TAF in adults with established virological suppression at 24 months.

## Methods

### Study population and design

The present retrospective real-world study included 326 virologically suppressed PLWH who switched from the EFV/TDF/3TC regimen to the DTG/3TC or B/F/TAF regimen between July 13, 2021, and October 19, 2024, at Hangzhou Xixi Hospital, the largest dedicated AIDS hospital in Zhejiang Province, China. The inclusion criteria were as follows: (1) receiving ART treatment with the EFV/TDF/3TC regimen for at least 12 months; (2) plasma HIV RNA <200 copies/mL for at least 12 months; and (3) age ≥18 years. The exclusion criteria were as follows: (1) Pregnancy or lactation; (2) resistance mutations to study drugs, previous therapy with study drugs; (3) allergic history or high degree of sensitivity to any component or auxiliary material of the research drug; (4) chronic hepatitis B or untreated hepatitis C; (5) patients with concomitant malignant tumors, and (6) individuals with a history of drug abuse. Application of these criteria resulted in the exclusion of twelve participants: six because of concomitant malignant tumors and six owing to a history of drug abuse. The remaining 326 PLWH received prior ART treatment with the EFV/TDF/3TC regimen for at least 12 months after HIV diagnosis. According to the different reasons and recommended usage guidelines for regimen switch, 71 PLWH switched to the DTG/3TC regimen, and 255 PLWH switched to the B/F/TAF regimen.

### Data collection

**FIGURE 1:**
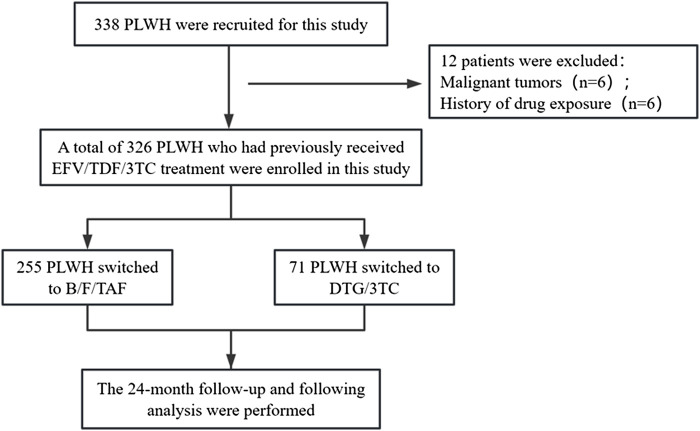
Study flow diagram. A total of 326 PLWH were included in the study. Abbreviations: PLWH, people living with HIV; B/F/TAF, bictegravir/emtricitabine/tenofovir alafenamide; DTG/3TC, dolutegravir/lamivudine.

Baseline demographic data and clinical variables were collected from the electronic medical record (EMR) management system. The following data were collected: age, sex, weight, BMI, marital status, transmission route, CD4^+^ T cell counts, educational background, occupation, monthly income, smoking status, drinking status, high blood pressure (HBP) status and ART therapy time. Weight, TG, TC, GLU, and SUA were collected at months 0 and 24. The study flowchart is shown in Figure 1.

### Outcomes

The primary outcomes were the change in HTG, HTC, HGLU, HUA, and the proportion of participants with >5% weight change at 24 months (Ryan et al., 2025). Key secondary outcomes were the trend and associated factors of TG, TC, GLU, SUA in total PLWH and with hypertension over the 24-month period. Other secondary endpoints were the changes in weight and treatment differences in patients with >5% weight gain at month 48.

### Diagnostic standards

Dyslipidemia (high TG or high TC) was defined as an abnormally high TG level (>1.7 mmol/L) or TC level (>5.2 mmol/L). Dysglycemia was defined as an abnormally high blood GLU level (fasting glucose level ≥5.6 mmol/L) (Ruiz-Algueró et al., 2022). An elevated BMI (overweight or obesity) was defined as BMI ≥25 kg/m^2^ for the purpose of this analysis (Yang et al., 2020). The definition of hyperuricemia (HUA) was based on the concentration of uric acid (UA) in serum, in which HUA was defined as an SUA level exceeding 420 μmol/L (World Health Organization, 1997; Dalbeth et al., 2021).

### Statistical analysis

All statistical analyses were performed using SPSS (v.25.0). The normally distributed continuous variables are presented as means with standard deviations (SDs) and were compared using t-tests (paired or unpaired). The non-normally distributed variables are presented as medians (Ms) and interquartile ranges (IQRs; 1st quartile and 3rd quartile), and they were compared using nonparametric Wilcoxon signed-rank tests (paired or unpaired). The categorical variables are presented as numbers and percentages. The proportions were compared using Pearson’s Chi-square test. All graphs were created using GraphPad Prism 8 software (GraphPad Software Inc., San Diego, CA, United States) (Zhang et al., 2025). A Generalized Linear Mixed Model (GLMM) was used for multivariable analysis of longitudinal changes in metabolic parameters, adjusting for time point, treatment group, age, smoking status, drinking status, hypertension, and baseline BMI. Logistic regression was used to assess treatment differences in patients with >5% weight gain at month 24. All reported levels of statistical significance were two-sided, and *p* values less than 0.05 were considered to indicate statistical significance.

## Results

326 PLWH received switching regimens to BF/TAF (n = 255) and DTG/3TC (n = 71; Figure 1). One patient (1.4%) in the DTG/3TC group met the criterion for virologic failure, with an HIV-1 RNA level >200 copies/mL at month 24. 319 (97.9%) of 326 participants were male and 7 (2.1%) were female. Mean age was 34.9 ± 10.2 years. Mean duration of previous antiretroviral therapy were 4.7 ± 2.1 years. Mean BMI was 22.3 ± 2.9 kg/m^2^. The primary indication for regimen switching was treatment-related side effects (113 cases; 34.7% Table 1).

**TABLE 1:** Baseline characteristics of all participants (n = 326).

Characteristics	Values
Age, years (before switching), (Mean ± SD)	34.9 ± 10.2
Sex, n (%)	
Male	319 (97.9)
Female	7 (2.1)
Marital status, n (%)	
Unmarried	261 (80.1)
Married	44 (13.5)
Divorced or widowed	21 (6.4)
Transmission route, n (%)	
Heterosexual contact	52 (16)
Homosexual contact	269 (82.5)
Others	5 (1.5)
CD4^+^T, cells/μL (before switching), n (%)	
≥200 cells/μL	317 (97.2)
<200 cells/μL	9 (2.8)
HIV-RNA, copies/mL (before switching), n (%)	
<50 copies/mL	317 (97.2)
50–200 copies/mL	9 (2.8)
Educational level, n (%)	
High school or below	49 (15)
Junior college	89 (27.3)
Undergraduate or above	188 (57.7)
Occupation, n (%)	
Civil servants and professionals	108 (33.1)
Others	218 (66.9)
Monthly income, n (%)	
>CNY 10,000	144 (44.2)
≤CNY 10,000	182 (55.8)
Smoking, n (%)	
Yes	109 (33.4)
No	217 (66.6)
Drinking, n (%)	
Yes	164 (50.3)
No	162 (49.7)
HBP, n (%)	
Yes	96 (29.4)
No	230 (70.6)
BMI, kg/m^2^ (before switching), (Mean ± SD)	22.3 ± 2.9
Drug side effects (Reasons for switching ART), n (%)	
Yes	113 (34.7)
No	213 (65.3)
ART time, years (before switching), (Mean ± SD)	4.7 ± 2.1
ART regimens, n (%)	
B/F/TAF	255 (78.2)
DTG/3TC	71 (21.8)
Lipid-lowering agents (before switching), n (%)	
Yes	2 (0.6)
No	324 (99.4)

Abbreviations: CD4^+^ T, CD4^−^positive T cells; CNY, Chinese Yuan; HBP, high blood pressure; BMI, body mass index; ART, antiretroviral therapy; SD, standard deviation; B/F/TAF, bictegravir/emtricitabine/tenofovir alafenamide; DTG/3TC, dolutegravir/lamivudine.

There was 1 patient in each group continued on regular lipid-lowering therapy both before and after regimen switch. Two patients initiated lipid-lowering therapy and one initiated SUA–lowering therapy after switching (in the B/F/TAF group). One patient (in the DTG/3TC group) initiated lipid-lowering therapy after switching. There were no differences in baseline characteristics between groups (all *p* > 0.05; Table 2; Supplementary Table S1). There were no statistically significant differences between B/F/TAF and DTG/3TC groups in HTG (93 [42.3%] vs. 20 [41.7%]), HTC (59 [26.8%] vs. 13 [27.1%]), HGLU (53 [24.1%] vs. 17 [35.4%]), or HUA (85 [35.7%] vs. 24 [38.1%]) at month 24 (all P > 0.05; Table 2).

**TABLE 2:** Comparison of the abnormal metabolic indexes between B/F/TAF and DTG/3TC groups.

Variables	M0			M24		
	B/F/TAF	DTG/3TC	*P* value	B/F/TAF	DTG/3TC	*P* value
HTG, n (%)						
Yes	91 (35.7)	21 (29.6)	0.338	93 (42.3)	20 (41.7)	0.939
No	164 (64.3)	50 (70.4)		127 (57.7)	28 (58.3)	
HTC, n (%)						
Yes	47 (18.4)	10 (14.1)	0.394	59 (26.8)	13 (27.1)	0.970
No	208 (81.6)	61 (85.9)		161 (73.2)	35 (72.9)	
HGLU, n (%)						
Yes	90 (35.3)	26 (36.6)	0.837	53 (24.1)	17 (35.4)	0.106
No	165 (64.7)	45 (63.4)		167 (75.9)	31 (64.6)	
HUA, n (%)						
Yes	49 (19.2)	16 (22.5)	0.536	85 (35.7)	24 (38.1)	0.727
No	206 (80.8)	55 (77.5)		153 (64.3)	39 (61.9)	

Abbreviations: M0, baseline; M24, post-24-month. HTG, hypertriglyceridemia; HTC, hypercholesterolemia; HGLU, hyperglycemia; HUA, hyperuricemia.

**FIGURE 2:**
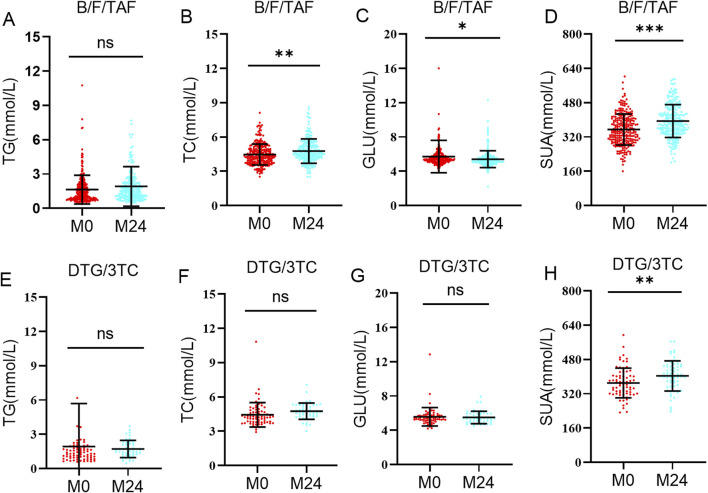
Changes in metabolic indicators from baseline to 24 months. Levels of triglyceride **(A,E)**, total cholesterol **(B,F)**, glucose **(C,G)**, and serum uric acid **(D,H)** in the B/F/TAF group and DTG/3TC group at baseline and 24 months. Statistical significance is represented by *P < 0.05, **P < 0.01, and ***P < 0.001. No statistically significant difference between groups at any time point is indicated by “ns” (non-significant, *P* > 0.05). Abbreviations: TC, total cholesterol; TG, triglyceride; GLU, glucose; SUA, serum uric acid.

After adjustment for potential confounders, the GLMM analysis showed that the trajectories of TG, TC, GLU, and SUA did not differ significantly between groups over time (all P > 0.05, Table 3). From baseline to month 24, switching to B/F/TAF exhibited significant increases in TC (P = 0.001; Figure 2B; Supplementary Table S2) and SUA (P < 0.001; Figure 2D), a decrease in GLU (P = 0.026; Figure 2C), and a borderline increase in TG (P = 0.053; Figure 2A). Switching to DTG/3TC showed a pronounced increase only in SUA (P = 0.006; Figure 2H), while TG, TC, and GLU levels remained stable (all P > 0.05; Figures 2E–G).

**TABLE 3:** Multivariable analysis of factors associated with metabolic indicators (TG, TC, GLU, SUA) in PLWH (n = 326).

Variables	TG		TC		GLU		SUA	
	*β* value (95% *CI*)	*P* value	*β* value (95% *CI*)	*P* value	*β* Value (95% *CI*)	*P* value	*β* value (95% *CI*)	*P* value
Intercept	1.475 (1.171–1.779)	<0.001	4.351 (4.202–4.499)	<0.001	5.486 (5.260–5.712)	<0.001	*346.098 (335.414 ∼ 356.782)*	<0.001
Time point								
M24 vs. M0	0.168 (−0.128–0.464)	0.265	0.292 (0.135–0.449)	<0.001	−0.273 (−0.487∼ −0.058)	0.013	−5.549 (−16.492–5.394)	0.320
ART regimens								
DTG/3TC vs. B/F/TAF	−0.014 (−0.389–0.360)	0.940	−0.055 (0.250–0.140)	0.580	−0.082 (−0.325–0.161)	0.509	12.122 (−1.708–25.951)	0.086
Age, years (before switching)								
≥50 vs. < 50	0.025 (−0.513–0.564)	0.926	−0.061 (−0.347–0.225)	0.676	0.394 (0.052–0.736)	0.024	−59.565 (−79.749∼ −39.381)	<0.001
Smoking								
Yes vs. No	0.424 (0.091–0.756)	0.013	0.203 (0.027–0.378)	0.024	0.094 (−0.119–0.307)	0.385	−5.967 (−18.364–6.429)	0.345
Drinking								
Yes vs. No	−0.022 (−0.339–0.296)	0.894	−0.145 (−0.313–0.022)	0.088	−0.088 (−0.291–0.116)	0.398	8.593 (3.227–20.412)	0.154
HBP								
Yes vs. No	0.162 (−0.167–0.490)	0.334	0.304 (0.130–0.479)	0.001	0.308 (0.099–0.517)	0.004	16.888 (4.604–29.171)	0.007
BMI, (kg/m^2^) before switching								
≥25 vs. < 25	0.316 (−0.083–0.715)	0.121	0.270 (0.059–0.481)	0.012	0.632 (0.377–0.886)	<0.001	42.468 (27.579–57.358)	<0.001

Abbreviations: M0, baseline; M12, post-12-month; M24, post-24-month. ART, antiretroviral therapy; B/F/TAF, bictegravir/emtricitabine/tenofovir alafenamide; DTG/3TC, dolutegravir/lamivudine; HBP, high blood pressure; BMI, body mass index.

The proportion of participants with >5% weight gain at 24 months was 99 of 255 (38.8%) in the B/F/TAF group and 33 of 71 (46.5%) in the DTG/3TC group (adjusted odds ratio [OR] for the effect of treatment 1·360 [95% CI 0.790–2.341]; p = 0·267; Table 4). Drinking was a statistically significant associated factor (adjusted odds ratio [OR] for the effect of treatment 1·921 [95% CI 1.182–3.122]; p = 0·008; Table 4).

**TABLE 4:** Multivariate binary logistic regression analysis of factors associated with >5% weight gain at 24 Months post-switch (n = 326).

Variables	Or (95% CI)	*P* value
ART regimens	1.360 (0.790–2.341)	0.267
DTG/3TC vs. B/F/TAF	​	​
Age, years (before switching)	1.298 (0.553–3.042)	0.549
≥50 vs. < 50	​	​
Smoking	0.967 (0.583–1.603)	0.895
Yes vs. No	​	​
Drinking	1.921 (1.182–3.122)	0.008
Yes vs. No	​	​
Hypertension	0.838 (0.501–1.404)	0.503
Yes vs. No	​	​
BMI, kg/m^2^ (before switching)	0.692 (0.367–1.306)	0.256
≥25 vs. < 25	​	​

Abbreviations: ART, antiretroviral therapy; B/F/TAF, bictegravir/emtricitabine/tenofovir alafenamide; DTG/3TC, dolutegravir/lamivudine; BMI, body mass index.

**FIGURE 3:**
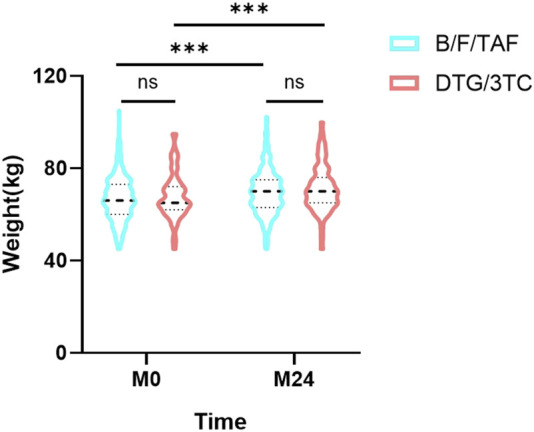
Changes in body weight from baseline to 24 months. Comparison of body weight in the B/F/TAF group and DTG/3TC group at baseline (M0) and after 24 months of follow-up (M24). No statistically significant difference between groups at any time point is indicated by “ns” (non-significant, *P* > 0.05). Abbreviations: B/F/TAF, bictegravir/emtricitabine/tenofovir alafenamide; DTG/3TC, dolutegravir/lamivudine.

There were no differences in weight between groups at baseline (66.9 ± 9.6 kg vs. 67.9 ± 9.9 kg, P = 0.442) or at month 24 (69.4 ± 9.6 kg vs. 71.2 ± 10.3 kg, P = 0.170). Weight changes from baseline all increased statistically significantly in B/F/TAF (66.9 ± 9.6 kg vs. 69.4 ± 9.6 kg, P < 0.001) and DTG/3TC group (67.9 ± 9.9 kg vs. 71.2 ± 10.3 kg, P < 0.001; Supplementary Table S3; Figure 3).

In the multivariable GLMM analysis, switching regimens was associated with an increasing trend in TC (P < 0.001) and a decreasing trend in GLU (P = 0.013). Independent associated factors included smoking for TG (P = 0.013); smoking, hypertension, and BMI ≥25 for TC (all P < 0.05); age ≥50, hypertension, and BMI ≥25 for GLU (all P < 0.01); and age ≥50, hypertension, and BMI ≥25 for SUA (all P < 0.01) (Table 3).

In PLWH with hypertension, baseline characteristics were generally comparable between those switching to B/F/TAF (n = 71) and those switching to DTG/3TC (n = 25) (Supplementary Table S4), with no significant differences in demographics, clinical parameters, or baseline metabolic markers (all P > 0.05) except for drinking status (P = 0.019). After 24 months of treatment, the two groups continued to show no statistically significant differences in the prevalence of HTG (34 [52.3%] vs. 7 [36.8%]), HTC (21 [32.3%] vs. 5 [26.3%]), HGLU (24 [36.9%] vs. 7 [36.8%]), or HUA (29 [43.9%] vs. 14 [60.9%]) (all P > 0.05; Table 5). After adjustment for potential confounders, the GLMM analysis showed that the trajectories of TG, TC, GLU, and SUA did not differ significantly between groups over time (all *P* > 0.05; Supplementary Table S5).

**TABLE 5:** Comparison of abnormal metabolic indexes between B/F/TAF and DTG/3TC regimens in PLWH with hypertension.

Variables	M0			M24		
	B/F/TAF	DTG/3TC	*P* value	B/F/TAF	DTG/3TC	*P* value
HTG, n (%)						
Yes	32 (45.1)	13 (52.0)	0.550	34 (52.3)	7 (36.8)	0.235
No	39 (54.9)	12 (48.0)		31 (47.7)	12 (63.2)	
HTC, n (%)						
Yes	21 (29.6)	5 (20.0)	0.354	21 (32.3)	5 (26.3)	0.619
No	50 (70.4)	20 (80.0)		44 (67.7)	14 (73.7)	
HGLU, n (%)						
Yes	38 (53.5)	12 (48.0)	0.635	24 (36.9)	7 (36.8)	0.995
No	33 (46.5)	13 (52.0)		41 (63.1)	12 (63.2)	
HUA, n (%)						
Yes	19 (26.8)	10 (40.0)	0.215	29 (43.9)	14 (60.9)	0.162
No	52 (73.2)	15 (60.0)		37 (56.1)	9 (39.1)	

Abbreviations: HTG, hypertriglyceridemia; HTC, hypercholesterolemia; HGLU, hyperglycemia; HUA, hyperuricemia.

## Discussion

Both in the overall cohort and in the subgroup with hypertension, switching to B/F/TAF and DTG/3TC groups revealed no significant differences in the longitudinal trends of TG, TC, GLU, and SUA levels. The incidence of metabolic abnormalities did not differ significantly between the two groups at M0 or at M24. These results are partially consistent to the PASO DOBLE study, which reported comparable virologic efficacy and no significant between-group differences in lipid changes at 48 weeks (Ryan et al., 2025). However, switching to B/F/TAF had statistically significant TC and SUA increase at M24. This finding is consistent with the broader observation that INSTI-based regimens can influence metabolic parameters including lipids and SUA (Wang et al., 2023), and aligns specifically with previous studies documenting increases in TC after switching from TDF- to TAF-based regimens (Waritu et al., 2024; Plum et al., 2021). The observed increase in TC is mechanistically understood to be associated with the discontinuation of TDF, which is known to exert a lipid-lowering effect (Surial et al., 2021). Consequently, switching to a TAF-containing regimen like B/F/TAF may result in a “rebound” increase in lipid levels. This is corroborated by the study of Martínez-Sanz J et al., which specifically reported increased TC after switching from TDF to TAF (Fabbiani et al., 2011). Therefore, regular screening of serum lipid profiles is recommended for PLWH receiving TAF-containing regimens like B/F/TAF. In addition, switching to B/F/TAF showed a statistically significant GLU decreasing at 24 months, which is consistent with the findings of Shi et al. (Martínez-Sanz et al., 2023). This phenomenon may be related to the reversal of mitochondrial toxicity or insulin resistance potentially induced by EFV prior to the switch (Shi et al., 2025; Dave et al., 2011; Lagathu et al., 2019). Thus, switching to the B/F/TAF regimen may offer additional benefits to virologically suppressed PLWH with concurrent hyperglycemia (Karamchand et al., 2016).

Notably, we observed significant weight gain over 24 months in both groups, with a mean increase of 2.5 kg in the B/F/TAF group and 3.3 kg in the DTG/3TC group. Clinically significant weight gain (>5%) occurred in approximately one-fifth to one-third of participants, consistent with reports of weight gain associated with modern INSTI-based regimens (Waritu et al., 2024; Plum et al., 2021; Daar et al., 2025). However, in contrast to the PASO DOBLE trial, which reported a statistically greater weight gain and a higher proportion of participants with >5% weight gain in the B/F/TAF group compared to the DTG/3TC group at 48 weeks (Ryan et al., 2025), our adjusted analysis found no significant difference in the risk of >5% weight gain between the B/F/TAF and DTG/3TC groups over 24 months. This discrepancy regarding weight gain outcomes highlights the potential influence of study population characteristics (e.g., baseline regimen composition, regional/ethnic factors, follow-up duration) and the interplay between the specific drugs being discontinued and those newly introduced.

With regard to associated factors for metabolic parameter trends, our multivariable GLMM identified smoking as a significant predictor for increased TG and TC levels, aligning with other research (Bourgi et al., 2020). Given the effects of smoking on lipid levels and the contribution of smoking to the increased risk of CVD endpoints among PLWH, smoking cessation efforts should be made a priority in HIV care. Additionally, our binary logistic regression analysis found that regular alcohol consumption was independently associated with >5% weight gain, highlighting another modifiable lifestyle factor. Furthermore, hypertension and a baseline BMI ≥25 kg/m^2^ were identified as associated factors for increased TC, GLU, and SUA levels, while age ≥50 years was a associated factor for increased GLU and SUA. Studies from Ethiopia, Tanzania, and other regions have reported a significant association between BMI ≥25 kg/m^2^ and elevated TC levels (Achila et al., 2022; Fiseha et al., 2021). Aging, HIV-related inflammation, ART duration, and endothelial dysfunction are recognized drivers of hypertension in PLWH (Muya and Kamuhabwa, 2019), highlighting the complex interplay among hypertension, obesity, and metabolic disorders in this population. This underscores the necessity of managing comorbidities and lifestyle factors to mitigate the risk of metabolic syndrome (García-Ruiz de Morales et al., 2025).

Our findings hold important clinical implications. They confirm modifiable risk factors (smoking, alcohol consumption, obesity, hypertension) for metabolic abnormalities and weight gain during ART, providing clear targets for clinical screening and intervention. In the context of regimen switching, the comparable metabolic and weight-gain profile of DTG/3TC and B/F/TAF in our study supports individualized treatment selection. The potential for differential weight gain, as suggested by trials like PASO DOBLE, should be considered alongside patient-specific factors such as baseline regimen (particularly TDF or ABC use), comorbidities, pill burden preference, and cost.

This study has certain limitations. Firstly, the sample size of the DTG/3TC group was relatively small (n = 71), which may have limited the statistical power for some subgroup analyses and could explain the lack of a detected difference in weight gain compared to the PASO DOBLE trial. Secondly, the analysis did not include the correlation between drug exposure (e.g., plasma drug concentrations) and metabolic indicators. Thirdly, our study explicitly excluded patients with chronic hepatitis B virus (HBV) coinfection to isolate the metabolic effects of the antiretroviral switch. Consequently, our findings are not directly generalizable to the important population of HIV/HBV-coinfected individuals, for whom HBV suppression must remain the primary determinant of regimen selection. Finally, the lack of objective assessment of body fat distribution (e.g., visceral adiposity) is a constraint. Future studies with larger sample sizes, longer follow-up durations, and the integration of pharmacogenomic and metabolomic technologies are warranted to further elucidate the mechanisms underlying the metabolic and weight effects of different ART regimens and to clarify the contextual factors that influence these outcomes.

## Conclusion

In this real-world study, virologically suppressed PLWH switching from EFV/TDF/3TC to either DTG/3TC or B/F/TAF exhibited comparable long-term metabolic changes over 24 months. Both regimens similarly increased weight, total cholesterol, and serum uric acid, with no significant intergroup differences. Traditional modifiable risk factors (including smoking, hypertension, elevated BMI, and alcohol use) were significantly linked to adverse metabolic outcomes. Therefore, the metabolic profile of TAF should not dominate the choice between two-drug (DTG/3TC) and three-drug (B/F/TAF) ART. Clinical decisions ought to be individualized, balancing regimen efficacy and tolerability with active management of patient-specific metabolic risks.

## Data Availability

The original contributions presented in the study are included in the article/Supplementary Material, further inquiries can be directed to the corresponding authors.
