# Supplementary material for: Metabolic impact of switching from EFV/TDF/FTC to two INSTI-Based regimens (B/F/TAF vs. DTG/3TC): a real-world study in virologically suppressed individuals
Source: Front Pharmacol. 2026 Mar 31;17:1753242. doi: 10.3389/fphar.2026.1753242 (PMC13076537; doi:10.3389/fphar.2026.1753242)
Supplement: Supplementary file 1 [file Table1.docx]

Table S1. Comparison of baseline characteristics between B/F/TAF and DTG/3TC groups.

| Characteristics | B/F/TAF  (n=255) | DTG/3TC  (n=71) | *P* value |
| --- | --- | --- | --- |
| Age, years(before switching),  n(%) |  |  |  |
| <50 | 235(92.2) | 64(90.1) | 0.586 |
| ≥50 | 20(7.8) | 7(9.9) |  |
| Sex,n(%) |  |  |  |
| Male | 249(97.6) | 70(98.6) | 0.982 |
| Female | 6(2.4) | 1(1.4) |  |
| Marital status, n (%) |  |  |  |
| Unmarried | 205(80.4) | 56(78.9) | 0.504 |
| Married | 32(12.5) | 12(16.9) |  |
| Divorced or widowed | 18(7.1) | 3(4.2) |  |
| Transmission route, n (%) |  |  |  |
| Heterosexual contact | 35(13.7) | 17(23.9) | 0.104 |
| Homosexual contact | 216(84.7) | 53(74.6) |  |
| Others | 4(1.6) | 1(1.4) |  |
| CD4^+^T (cells/μL)  (before switching), n (%) |  |  |  |
| ≥200 cells/μL | 247(96.9) | 70(98.6) | 0.706 |
| <200 cells/μL | 8(3.1) | 1(1.4) |  |
| HIV-RNA, copies/mL (before switching), n (%) |  |  |  |
| <50 copies/mL | 250（98.0） | 67（94.4） | 0.207 |
| 50～200 copies/mL | 5（2.0） | 4（5.6） |  |
| Educational background, n (%) |  |  |  |
| High school or below | 35(13.7) | 14(19.7) | 0.399 |
| Junior college | 69(27.1) | 20(28.2) |  |
| Undergraduate or above | 151(59.2) | 37(52.1) |  |
| Occupation, n (%) |  |  |  |
| Civil servants & professionals | 91(35.7) | 17(23.9) | 0.063 |
| Others | 164(64.3) | 54(76.1) |  |
| Monthly income, n (%) |  |  |  |
| >CNY 10,000 | 114(44.7) | 30(42.3) | 0.713 |
| ≤CNY 10,000 | 141(55.3) | 41(57.7) |  |
| Smoking , n (%) |  |  |  |
| Yes | 86(33.7) | 23(32.4) | 0.833 |
| No | 169(66.3) | 48(67.6) |  |
| Drinking , n (%) |  |  |  |
| Yes | 124(48.6) | 40(56.3) | 0.250 |
| No | 131(51.4) | 31(43.7) |  |
| Hypertension, n (%) |  |  |  |
| Yes | 71(27.8) | 25(35.2) | 0.228 |
| No | 184(72.2) | 46(64.8) |  |
| Weight ( kg), , before switching | 66.9±9.6 | 67.9±9.9 | 0.442 |
| BMI ,(kg/m^2^)before switching ,  n (%) |  |  |  |
| <25.0 | 217（85.1） | 55（77.5） | 0.223 |
| 25.0～29.9 | 35（13.7） | 15（21.1） |  |
| ≥30.0 | 3（1.2） | 1（1.4） |  |
| ART therapy time, years (before switching), n (%) |  |  |  |
| 1～5 | 169(66.3) | 44(62) | 0.500 |
| 6～10 | 86(33.7) | 27(38) |  |
| TG (mmol/L), before switching | 1.3(0.8,2) | 1.3(1,1.8) | 0.872 |
| TC (mmol/L) , before switching | 4.4(3.8,5) | 4.2(3.8,4.7) | 0.476 |
| GLU (mmol/L) , before switching | 5.4(5.1,5.8) | 5.4(5.2,5.7) | 0.855 |
| SUA(mmol/L) , before switching | 354.8±72.5 | 370.3±69.2 | 0.107 |
| Lipid-lowering agents (before switching), n (%) |  |  | 0.389 |
| Yes | 1(0.4) | 1(1.4) |  |
| No | 254(99.6) | 70(98.6) |  |
|  |  |  |  |

Abbreviations: CD4+ T, CD4- positive T cells; CNY, Chinese Yuan ; HBP, high blood pressure; BMI, body mass index; ART, antiretroviral therapy; SD, standard deviation; B/F/TAF, bictegravir/emtricitabine/tenofovir alafenamide; DTG/3TC, dolutegravir/lamivudine. TC, total cholesterol; TG, triglyceride; GLU, glucose; SUA, serum uric acid.

Table S2. The changes in the levels of metabolic indicators among PLWH who switched to DTG/3TC or B/F/TAF regimens during the 24-month follow-up.

| Paramaters | M0 | M24 | *P* value |
| --- | --- | --- | --- |
| B/F/TAF |  |  |  |
| TG (mmol/L) | 1.3(0.8,2.0) | 1.5(1,2.2.0) | 0.053 |
| TC (mmol/L) | 4.4(3.8,5.0) | 4.6(4.1,5.3) | 0.001** |
| GLU (mmol/L) | 5.4(5.1,5.8) | 5.2(5.0,5.6) | 0.026* |
| SUA(mmol/L) | 354.8±72.5 | 394.4±76.7 | <0.001*** |
| DTG/3TC |  |  |  |
| TG (mmol/L) | 1.3(1.0,1.8) | 1.6(1.2,2.2) | 0.654 |
| TC (mmol/L) | 4.2(3.8,4.7) | 4.7(4.3,5.3) | 0.055 |
| GLU (mmol/L) | 5.4(5.2,5.7) | 5.4(5.0,5.9) | 0.633 |
| SUA(mmol/L) | 370.3±69.2 | 403.5±70.3 | 0.006** |

Note: **,*P*<0.05;**,*P*<0.01 ****P* < 0.001.

Abbreviations: M0, baseline; M12, post-12-month; M24, post-24-month; TC, total cholesterol; TG, triglyceride; GLU, glucose; SUA, serum uric acid.

**Table S3. Comparison of Weight (kg) at Baseline and 24 Months Between Treatment Groups**

| Time | B/F/TAF^a^ | DTG/3TC^b^ | *P* value |
| --- | --- | --- | --- |
| M0 | 66.9±9.6 | 67.9±9.9 | 0.442 |
| M24 | 69.4±9.6 | 71.2±10.3 | 0.170 |

Abbreviations: a, *P* < 0.001 for within-group change from M0 to M24 in the B/F/TAF group.

b, *P* < 0.001 for within-group change from M0 to M24 in the DTG/3TC group.

**Table S4.** Comparison of Baseline Characteristics Between B/F/TAF and DTG/3TC Groups in HIV - Comorbid Hypertension Population

| Characteristics | B/F/TAF  (n=71) | DTG/3TC  (n=25) | *P* value |
| --- | --- | --- | --- |
| Age, years (before switching),n (%) |  |  |  |
| <50 | 57(80.3) | 22(88.0) | 0.572 |
| ≥50 | 14(19.7) | 3(12.0) |  |
| Sex, n (%) |  |  |  |
| Male | 68(95.8) | 25(100.0) | 0.707 |
| Female | 3(4.2) | 0(0.0) |  |
| Marital status, n (%) |  |  |  |
| Unmarried | 44(62) | 18(72.0) | 0.785 |
| Married | 17(23.9) | 4(16.0) |  |
| Divorced or widowed | 10(14.1) | 3(12.0) |  |
| Transmission route, n (%) |  |  |  |
| Heterosexual contact | 17(23.9) | 6(24.0) | 0.681 |
| Homosexual contact | 53(74.6) | 18(72.0) |  |
| Others | 1(1.4) | 1(4.0) |  |
| CD4+T (cells/μL)(before switching), n (%) |  |  |  |
| ≥200 cells/μL | 69(97.2) | 25(100.0) | 0.973 |
| <200 cells/μL | 2(2.8) | 0(0.0) |  |
| HIV-RNA, copies/mL (before switching), n (%) |  |  |  |
| <50 copies/mL | 70（98.6） | 22（88.0） | 0.090 |
| 50～200 copies/mL | 1(1.4) | 3(12.0) |  |
| Educational background, n (%) |  |  |  |
| High school or below | 16(22.5) | 5(20.0) | 0.963 |
| Junior college | 17(23.9) | 6(24.0) |  |
| Undergraduate or above | 38(53.5) | 14(56.0) |  |
| Occupation, n (%) |  |  |  |
| Civil servants & professionals | 27(38) | 7(28.0) | 0.367 |
| Others | 44(62) | 18(72.0) |  |
| Monthly income, n (%) |  |  |  |
| >CNY 10,000 | 34(47.9) | 10(40.0) | 0.496 |
| ≤CNY 10,000 | 37(52.1) | 15(60.0) |  |
| Smoking , n (%) |  |  |  |
| No | 51(71.8) | 16(64.0) | 0.463 |
| Yes | 20(28.2) | 9(36.0) |  |
| Drinking , n (%) |  |  |  |
| No | 42(59.2) | 8(32.0) | 0.019 |
| Yes | 29(40.8) | 17(68.0) |  |
| Weight,(kg) | 70.5±10.2 | 72.5±11.3 | 0.397 |
| BMI ,(kg/m2)before switching , n (%) |  |  |  |
| <25.0 | 55（77.5） | 17（68.0） | 0.507 |
| 25.0～29.9 | 14（19.7） | 7（28.0） |  |
| ≥30.0 | 2（2.8） | 1（4.0） |  |
| ART therapy time, years (before switching), n (%) |  |  |  |
| 1～5 | 50(70.4) | 17(68.0) | 0.821 |
| 6～10 | 21(29.6) | 8(32.0) |  |
| TG (mmol/L), before switching | 1.6(0.9,2.6) | 1.7(1.1,2.3) | 0.967 |
| TC (mmol/L) , before switching | 4.6(4.1,5.3) | 4.5(3.8,4.5) | 0.447 |
| GLU (mmol/L) , before switching | 5.6(5.4,6.3) | 5.6(5.3,5.8) | 0.15 |
| SUA(mmol/L) , before switching | 360.6±76.6 | 390.1±83.5 | 0.109 |

Abbreviations: CD4+ T, CD4- positive T cells; CNY, Chinese Yuan ; HBP, high blood pressure; BMI, body mass index; ART, antiretroviral therapy; SD, standard deviation; B/F/TAF, bictegravir/emtricitabine/tenofovir alafenamide; DTG/3TC, dolutegravir/lamivudine. TC, total cholesterol; TG, triglyceride; GLU, glucose; SUA, serum uric acid.

Table S5. Multivariable Analysis of Factors Associated with Metabolic Indicators (TG, TC, GLU, SUA) in PLWH with Hypertension(n=96)

| Variables | TG | | TC | | GLU | | SUA | |
| --- | --- | --- | --- | --- | --- | --- | --- | --- |
|  | *β* value  (95% *CI*) | *P* value | *β* value (95% *CI*) | *P* value | *β* value (95% *CI*) | *P* value | β value (95% *CI*) | *P* value |
| Intercept | 1.815  (1.448~ 2.182) | <0.001 | 4.714  (4.418~  5.009) | <0.001 | 6.055  (5.447~  6.663) | <0.001 | 373.725  (352.659~394.791) | <0.001 |
| Time point |  |  |  |  |  |  |  |  |
| M24 vs M0 | -0.066  (0.427~  0.295) | 0.719 | 0.299  (0.028~  0.627) | 0.073 | -0.485  (1.073~  0.103) | 0.105 | -4.025  (25.540~  17.490) | 0.712 |
| ART regimens |  |  |  |  |  |  |  |  |
| DTG/3TC vs B/F/TAF | -0.255  (0.695~  0.185) | 0.254 | -0.235  (0.615~  0.146) | 0.225 | -0.206  (0.628~  0.216) | 0.336 | 24.649  (1.523~  50.821) | 0.065 |
| Age, years  (before switching) |  |  |  |  |  |  |  |  |
| ≥50 vs <50 | 0.088  (0.395~  0.572) | 0.719 | -0.207  (0.631~  0.217) | 0.336 | 0.577  (0.125~  1.029) | 0.013 | -76.863  (105.798~-47.929) | <0.001 |
| Smoking |  |  |  |  |  |  |  |  |
| Yes vs No | 0.437  (0.021~  0.853) | 0.039 | 0.431  (0.068~  0.794) | 0.02 | 0.380  (0.012~  0.772) | 0.057 | -0.128  (24.948~  24.692) | 0.992 |
| Drinking |  |  |  |  |  |  |  |  |
| Yes vs No | 0.014  (0.385~  0.414) | 0.944 | -0.255  (0.604~  0.094) | 0.151 | -0.410  (0.786~  -0.033) | 0.033 | -14.846  (38.712~  9.021) | 0.221 |
| BMI,(kg/m^2^) before switching |  |  |  |  |  |  |  |  |
| ≥25 vs <25 | 0.256  (0.169~  0.682) | 0.236 | 0.243  (0.129~  0.616) | 0.199 | 0.516  (0.118~  0.915) | 0.011 | 35.668  (10.232~  61.105) | 0.006 |

Abbreviations: M0, baseline; M12, post-12-month; M24, post-24-month. ART, antiretroviral therapy; B/F/TAF, bictegravir/emtricitabine/tenofovir alafenamide; DTG/3TC, dolutegravir/lamivudine; HBP, high blood pressure; BMI, body mass index
